# A novel cloud-based artificial intelligence for real-time detection of colorectal neoplasia – a randomized controlled trial (EAGLE)

**DOI:** 10.1038/s41746-025-02270-1

**Published:** 2025-12-26

**Authors:** Rawen Kader, Cesare Hassan, Ángel Lanas, Marcin Romańczyk, Tomasz Romańczyk, Bronisław Kotowski, Carlos Sostres Homedes, Benedetto Mangiavillano, Giacomo Bonanno, Laurence B. Lovat, Michał Kamiński, Siegbert Faiss, Alessandro Repici

**Affiliations:** 1 Division of Surgery and Interventional Sciences, University College London, London, UK; 2 Department of Biomedical Sciences, Humanitas University, Pieve Emanuele, Italy; 3 IRCCS Humanitas Research Hospital, Rozzano, Italy; 4 Instituto de Investigación Sanitaria Aragón, Instituto Aragonés de Ciencias de La Salud, CIBEREHD, Zaragoza, Spain; 5 Gastroenterology, Hospital Clínico Universitario de Zaragoza, Instituto de Investigación Sanitaria Aragón, Universidad de Zaragoza, CIBEREHD, Zaragoza, Spain; 6 Department of Gastroenterology, Faculty of Medicine, Academy of Silesia, Katowice, Poland; 7 Endoterapia, H-T Centrum Medyczne, Tychy, Poland; 8 Department of Gastroenterological Oncology, The M. Sklodowska-Curie Memorial Cancer Center and Institute of Oncology, Warsaw, Poland; 9 Polish Foundation of Gastroenterology, Warsaw, Poland; 10 Gastrointestinal Endoscopy, Istituto Clinico Mater Domini Casa di Cura Privata SpA, Castellanza, Italy; 11 Endoscopy Unit, Humanitas Istituto Clinico Catanese, Catania, Italy; 12 Department of Oncological Gastroenterology, Maria Sklodowska-Curie National Research Institute of Oncology, Warsaw, Poland; 13 Department of Cancer Prevention, Maria Sklodowska-Curie National Research Institute of Oncology, Warsaw, Poland; 14 Department of Gastroenterology, Sana Klinikum Lichtenberg, Berlin, Germany

**Keywords:** Gastrointestinal cancer, Endoscopy, Colonoscopy

## Abstract

Previously, colorectal polyp computer-aided detection (CADe) systems required on-site high-performance hardware installations (e.g., FPGAs/GPUs), creating practical challenges to upgrades and tying hospitals to legacy hardware. Cloud-based CADe solutions overcome these constraints. Hospitals can use low-specification/low-cost hardware to stream data to the cloud for analysis, enabling frequent AI hardware and algorithm updates. Furthermore, existing CADe systems’ benefits are largely limited to smaller, less clinically relevant polyps ( < 10 mm). This parallel-group RCT evaluated a real-time cloud-deployed CADe-system trained on an enhanced dataset of clinically significant polyps (large polyps( ≥ 10 mm) and sessile-serrated-lesions(SSLs)). Patients from eight centers across four European countries (841 patients, 22 endoscopists) were randomized to standard or CADe-assisted colonoscopy. Co-primary endpoints were (1) superior Adenomas Per-Colonoscopy (APC), (2) non-inferior Positive Percent-Agreement (PPA) (proportion of resections confirmed as clinically relevant polyps). CADe improved (*p* < 0.05): APC (0.82 vs. 0.62, Ratio 1.33[95% CI 1.06–1.67]), adenoma detection-rate (43.2% vs. 35.9%), SSL (0.08 vs. 0.03, Ratio 3.30[95% CI 1.41–7.57]), and large polyp (0.12 vs. 0.05, Ratio 2.36[95% CI 1.33–4.17]) detection. PPA was non-inferior, and average cloud-network latency was 59.4 ms per minute, with 99.6% under the 100 ms threshold required for real-time use. This RCT demonstrates the feasibility and efficacy of a real-time cloud-based CADe system, with promising outcomes for clinically significant polyps (large polyps and SSLs). Future research should explore optimizing CADe systems' performance. ClinicalTrials.gov (NCT05730192[15/02/2023]).

## Introduction

Colorectal Cancer (CRC) is the third most common cancer and the second leading cause of cancer-related death worldwide^1,2^. Colonoscopy is the gold standard for CRC screening, enabling detection and removal of neoplastic polyps, including adenomas and sessile serrated lesions (SSLs)^3^. However, colonoscopy is not infallible, with miss-rates as high as 26% for adenomas and 27% for SSLs^4^. Studies show that polyp detection directly correlates with post-colonoscopy colorectal cancer (PCCRC) risk, with missed polyps mainly due to inadequate mucosal visualization and failure to recognize visible polyps^5–8^.

In recent years, there has been a rise in randomized controlled trials (RCT) assessing Computer-Aided Detection (CADe) systems to improve polyp detection. Meta-analyses of RCTs confirm CADe improves polyp detection; however, key limitations remain^9^. Existing CADe systems primarily improve detection of smaller polyps (<10 mm) but have shown limited effectiveness in improving detection of more clinically significant polyps, such as larger polyps (≥10 mm) and SSLs^9^. Meta-analysis of tandem colonoscopy demonstrates a miss-rate of 6% for large adenomas^4^, which is clinically relevant given that large-sized polyps are known to harbor a higher risk of malignant transformation^10^. SSLs are notoriously difficult to detect owing to their flat morphology and subtle endoscopic features^11,12^ and pose a disproportionately high PCCRC risk^13^.

A key limitation of traditional polyp CADe systems and studies has been the requirement to run the AI algorithm on-site in the hospital. This means that local, high-performance computer hardware is required, which can be costly, especially if it needs to be upgraded for future versions of the algorithms. Furthermore, software updates can be complex, time-consuming, and require a technician to attend in person. Cloud-based artificial intelligence (AI) systems represent a transformative shift, enabling real-time, high-performance computation without these constraints. Edge-cloud hybrid architectures allow seamless back-end algorithm updates, providing clinicians with instant access to the latest AI models, whilst also offering improved accessibility and scalability. This novel paradigm shift in AI endoscopy has been largely underexplored to date.

This study evaluates the clinical efficacy, safety, and technical feasibility of a novel cloud-native CADe system (‘CADDIE’), which was developed with an enriched training dataset of clinically significant polyps (large polyps and SSLs).

## Results

**Fig. 1:**
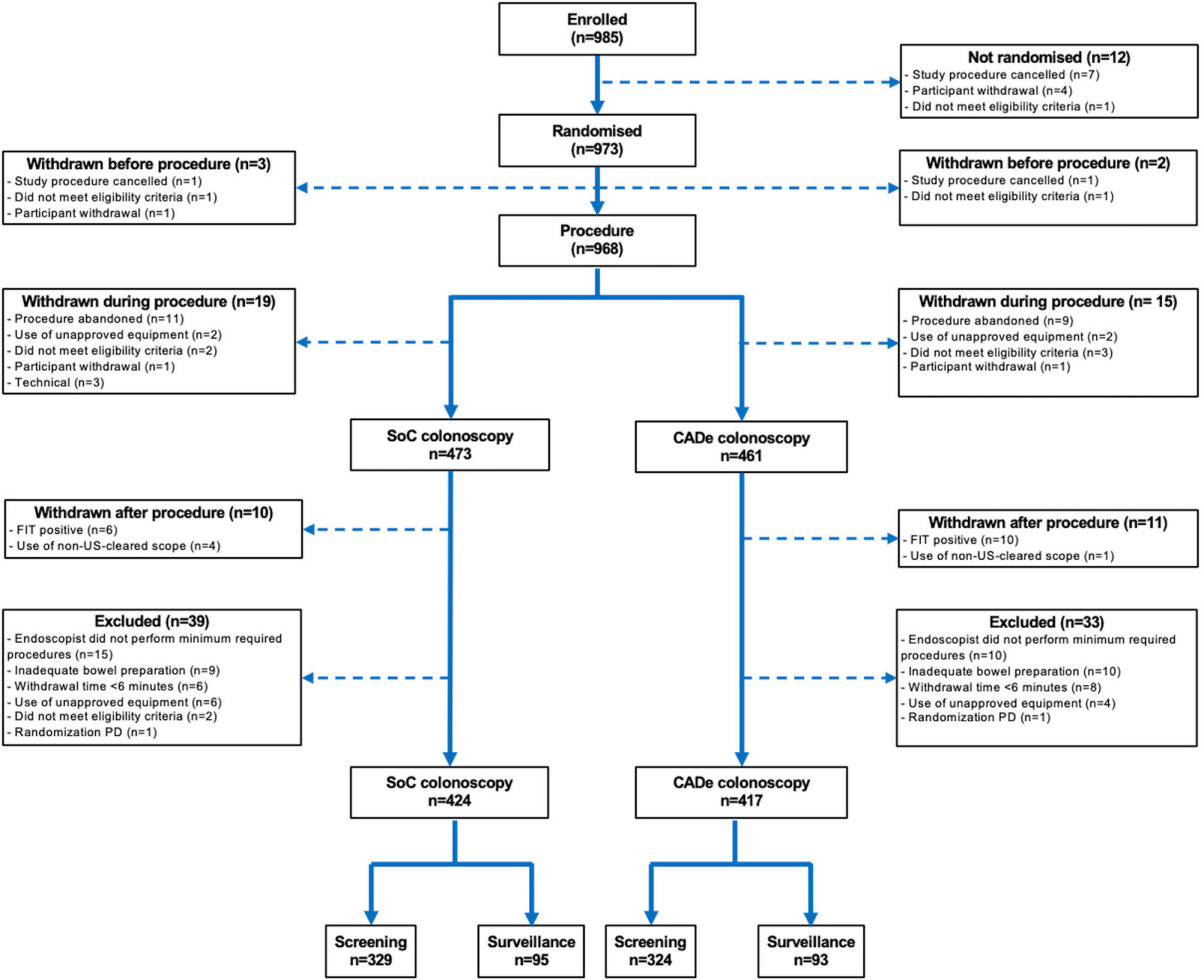
CONSORT recruitment flowchart. Seventeen patients were withdrawn before the colonoscopy, 12 of which were not randomized. Thirty-four patients were withdrawn during the procedure (20 procedures abandoned (16 due to inadequate bowel prep preventing intubation, one due to stenosis, one due to nausea, one due to hypoxia and one unknown), five identified during the procedure to not meet patients eligibility criteria, four procedures used unapproved endoscopes per-protocol, three technical issues (e.g. computer not connected to the endoscopy stack) and two patients withdrawals). Twenty-one completed procedures were excluded after it was identified post-procedurally to be a patient with positive fecal immunochemical test (FIT) or the procedure used CF-EZ1500DI endoscopes which were not cleared for use in the US by the end of the study. A further 72 were excluded. This included 25 procedures from endoscopists who failed to complete the minimum ten procedures required, 19 with inadequate bowel preparation (BBPS < 2 in any one of the three colonic segments), 14 with a withdrawal time below 6 min, ten due to the use of unapproved equipment (six unapproved scopes per-protocol, two performed withdrawal in TXI or NBI, two procedures used a different CADe system from ‘CADDIE’), two randomization errors where the procedure was not performed according to the randomized allocation, and two patients only identified at the end of the procedure to not meet patients eligibility criteria. Image created using Microsoft PowerPoint, version 16.103.2 (2025).

A total of 29 endoscopists were recruited, and 985 patients were invited, with 973 randomized between May and November 2023. Of those randomized, 132 patients were excluded (13.6%), as detailed in Fig. 1. In brief, these included the following reasons: (1) five withdrawn pre-procedure (e.g. ineligibility), (2) 34 withdrawn during the procedure (e.g. poor bowel preparation upon intubation), (3) 21 withdrawn post-procedure (e.g. identified post-procedure to be ineligible because FIT positive or the use of the CF-EZ1500DI endoscope which was not FDA-cleared by the end of the study), (4) 72 excluded due to inadequate quality procedures, endoscopists not meeting the minimum case requirement, or other protocol deviations. The proportion of withdrawn and excluded cases did not significantly differ between study arms (*X*^2^ (1, *n* = 973), *p* = 0.47), demonstrating balanced exclusions across arms.

The analyzed study population included 841 patients (417 CADe-arm, 424 SoC-arm), with 428 (50.9%) male, 412 (49.0%) female, and one (0.1%) was other, and a mean age was 58.5 ± 9.3 years. Screening accounted for 653 (77.7%) patients and 188 (22.3%) for surveillance. Endoscopic model (Supplementary Table 4) and procedural characteristics, including sedation and bowel preparation quality (Supplementary Table 5), were similar in both arms.

### Technical outcome measures

The total colonoscopy procedure time across the study was 31,151 min. The average cloud network latency was 59.4 ms and remained below 100 ms per minute for 99.6% of the procedural time (Supplementary Table 6). One procedure had an average that exceeded this threshold.

### Clinical outcome measures

Overall, 332 patients presented with at least one adenomatous lesion (39.5%), with a total of 552 adenomatous lesions. Primary endpoints (Table 1) showed a significantly higher APC in the CADe-arm compared to the SoC-arm (0.82 vs. 0.62, *p* = 0.01), with 33% more adenomas detected (Ratio 1.33 [95% CI 1.06–1.67]). For PPA, the CADe-arm demonstrated non-inferiority, with a numerically higher PPA (53.9% vs. 53.4%; 0.5%[−5.0%, ∞]), demonstrating no increase in unnecessary resections.

**Table 1:** Results of study endpoints

Outcome	Metric	Study arm	*N* patients	Total resections	Summary^a^	Difference^b^ (95% CI)	*p* value
Safety endpoint	PPA	SoC	424	517	276 (53.4%)	0	–
		CADe	417	700	377 (53.9%)	0.5% (−5.0%, ∞)^c^	
Adenoma detection metrics	APC (overall)	SoC	424	262	0.62 ± 1.19	1	**0.01**
		CADe	417	343	0.82 ± 1.40	1.33 (1.06, 1.67)	
	APC (distal)	SoC	424	121	0.29 ± 0.61	1	0.07
		CADe	417	158	0.38 ± 0.84	1.32 (0.98, 1.79)	
	APC (proximal)	SoC	424	141	0.33 ± 0.85	1	**0.05**
		CADe	417	185	0.44 ± 0.86	1.33 (1.00, 1.77)	
	ADR	SoC	424	–	152 (35.9%)	0	**0.03**
		CADe	417	–	180 (43.2%)	7.3% (0.7%, 13.9%)	
Neoplastic polyp detection metrics	NPPC	SoC	424	280	0.66 ± 1.20	1	**0.002**
		CADe	417	381	0.91 ± 1.48	1.39 (1.12, 1.73)	
	NSP-PC	SoC	424	18	0.04 ± 0.21	1	**0.03**
		CADe	417	38	0.09 ± 0.46	2.15 (1.07, 4.32)	
Polyps (all) detection metrics	PDR	SoC	424	–	225 (53.1%)	0	**0.04**
		CADe	417	–	251 (60.2%)	6.8 (0.3, 13.3)	
	PPC (overall)	SoC	424	451	1.06 ± 1.53	1	**0.001**
		CADe	417	589	1.41 ± 1.85	1.35 (1.13, 1.61)	
	PPC (distal)	SoC	424	238	0.56 ± 0.94	1	**0.008**
		CADe	417	315	0.76 ± 1.25	1.35 (1.08, 1.69)	
	PPC (proximal)	SoC	424	213	0.50 ± 0.97	1	**0.02**
		CADe	417	274	0.66 ± 1.07	1.31 (1.04, 1.65)	
SSL detection metric	SSL-PC	SoC	424	11	0.03 ± 0.16	1	**0.006**
		CADe	417	34	0.08 ± 0.45	3.30 (1.41, 7.571	

*APC* adenomas per colonoscopy, *ADR* adenoma detection rate, *NPPC* neoplastic polyps per colonoscopy, *PDR* polyp detection rate, *PPA* positive percent agreement, *PPC* polyps per colonoscopy, *NSP-PC* serrated neoplastic polyps per colonoscopy, *SSL-PC* sessile serrated lesions per colonoscopy.

^a^Summary statistics are mean ± standard deviation, median [inter-quartile range], or number (percentage).

^b^Group differences are ratio of values in CADe relative to SoC (continuous outcomes), or difference in percentages for adenoma detection rate.

^c^Percentage difference performed analyzed using one-sided 97.5% CI.

**Fig. 2:**
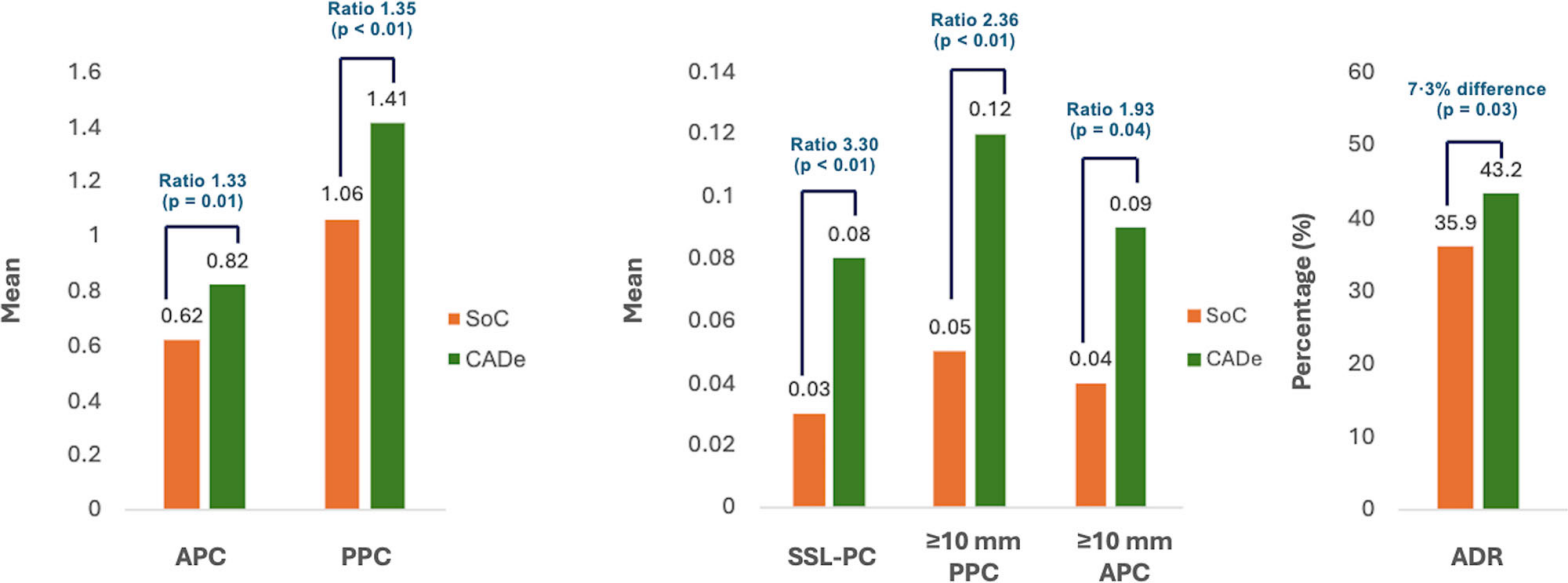
Graphical representation of the clinical outcomes in the CADe-arm and standard of care (SoC) arm. ADR adenoma detection-rate, APC adenomas per colonoscopy, PPC polyps per colonoscopy, SSL-PC sessile serrated lesions per colonoscopy. Image created using Microsoft PowerPoint version 16.103.2 and Microsoft Excel version 16.103.2 (2025).

Regarding secondary and exploratory endpoints, the CADe-arm demonstrated a significantly higher ADR (43.2% vs. 35.9%), with a 7.3% difference ([95% CI 0.7%–13.9%], *p* = 0.03) (Table 1 and Fig. 2). Mean polyp detection metrics significantly favored the CADe-arm, including SSL-PC (0.08 vs. 0.03; Ratio 3.30 [95% CI 1.41–7.57], *p* < 0.01), NPPC (0.91 vs. 0.66; Ratio 1.39 [95% CI 1.12–1.73], *p* < 0.01)), and PPC (1.41 vs. 1.06; Ratio 1.35 [95% CI 1.13–1.61], *p* = 0.001).

The CADe-arm demonstrated significantly higher detection of large adenomas (large APC 0.09 vs. 0.04; Ratio 1.93 [95% CI 1.03–3.62], *p* = 0.04), and large polyps (large PPC 0.12 vs. 0.05; Ratio 2.36 [95% CI 1.33–4.17], *p* < 0.01) (Fig. 2). Regarding morphology, the proportion of large polyps that were non-polypoid (38.0% vs. 27.3%) and large adenomas that were non-polypoid (22.9% vs. 15.8%) was higher in the CADe-arm compared to SoC-arm. Furthermore, large non-polypoid PPC was significantly higher in the CADe-arm (Ratio 3.34 (95% CI 1.10, 10.2, *p* = 0.03)), while large non-polypoid APC was numerically higher but not significant (Ratio 2.71 (95% CI 0.43–16.9, *p* = 0.29) (Supplementary Table 7). Results of polyp detection metrics stratified by site are available in Supplementary Table 8a–g.

Across all polyp sizes, detection was higher in the CADe-arm for both polypoid (mean PCC 0.74 vs. 0.57; Ratio 1.33, *p* = 0.02) and non-polypoid (mean PPC 0.53 vs. 0.40, Ratio 1.37, *p* = 0.03) polyps. Proximal adenomas and proximal polyps were also significantly higher in the CADe-arm (Table 1).

Total procedure time and CWT were significantly higher in the CADe-arm by 1 min, but the same for polyp negative procedures (Supplementary Table 5). Five AEs (three SoC-arm, two CADe-arm) were reported, all procedure-related and unrelated to the device. Events included patients with abdominal pain, gastrointestinal disorders, nausea, and hypoxia.

## Discussion

All prior studies in AI for endoscopy, including applications beyond polyp CADe, have relied on local hardware deployment, where the algorithms are hosted directly on-site in the hospital^14^. The EAGLE study addresses this limitation by being the first study to evaluate a cloud-based CADe deployment for real-time detection of colorectal neoplasia. Cloud-based AI algorithms have already proven effective and safe in various medical specialities^15^, such as the FDA-cleared EyeArt (Eyenuk) system, which detects more-than-mild diabetic retinopathy from fundus images, and BraveCX (Bering Limited), which analyses chest x-rays to identify critical findings such as pleural effusion and pneumothorax^16^^,17^. However, these cloud-based AI applications are designed for investigations that typically do not require real-time decision-making to guide an immediate intra-procedural therapy, as is the case with CADe in colonoscopy.

The cloud-based deployment of polyp CADe for colonoscopy requires a solution capable of processing high-volume image data, as modern endoscopy image processors can generate a frame rate of up to 50–60 images per second. To aid endoscopists in detecting polyps, CADe is required to process this data stream in real-time in order to alert the endoscopist to the polyp’s presence. The process includes transmitting the image data to the cloud, applying the algorithm, sending the results back to the hospital, and displaying the bounding-box results on the endoscopy screen. The speed of this process is influenced on the hospital IT network performance.

The EAGLE study confirms the technical feasibility of this workflow in endoscopy. The system was evaluated across eight centers in four countries in Eastern and Western Europe, providing a real-world and diverse testing environment. The average cloud network latency per minute across the study’s colonoscopy procedures (31,151 min) was 59.4 ms, with 99.6% of total procedural time below the 100 ms threshold. Across study centers, the average per-minute latencies that were recorded below the threshold ranged from 98.2% to 100.0%, highlighting the system’s generalizability and robustness despite variations in IT infrastructure. Importantly, no modifications to hospital IT infrastructure were necessary, as the system was integrated into existing network systems and clinical workflows. Furthermore, all procedures were performed during standard working hours, where network traffic is typically at its highest. The robust cloud network latency performance observed reflects the cloud-based system’s utilization of a highly optimized workflow, leveraging for ultra-low latency communication and minimal bandwidth usage, requiring only 8 mbps for upstream data transfer to the cloud, and 1 mbps for downstream data transfer.

It is important to consider the implications if latencies exceed this threshold. This occurred in 0.37% of the total study procedure time, and in one procedure, the average latency was above this threshold. The primary consequence of high cloud network latency is a lag in the visualization of detection boxes. However, the clinical results demonstrate non-inferiority in PPA, reflecting no increase in unnecessary resections, suggesting that the cloud-based CADe system does not negatively impact clinical decision-making. Irrespective of these results, it is also important for cloud-based solutions to have safeguard measures in place for when excess cloud network latency occurs in order to minimize disruption to routine clinical practice, such as disabling the display of the bounding box and alerting the user, as described in Methods. This enables the endoscopist to continue the procedure without CADe assistance.

Cloud-based deployment offers several advantages. The main advantage is the ability to provide endoscopists with continuous access to the latest CADe algorithm. Updates can be implemented centrally in the cloud on one deployment, allowing all users to benefit immediately without requiring on-site visits or potential replacement or reinstallation of hardware with updates. Given that the applications of AI in endoscopy are still in their infancy, several new applications for endoscopy are expected, such as detecting neoplasia in Barrett’s esophagus, endoscopic scoring of disease activity in IBD, and automating measurement of quality indicators such as withdrawal time, and more^18^^,^^19^. Furthermore, several changes to existing algorithms are also to be expected as the algorithms’ training datasets increase in size. This phenomenon has already been observed in the short time that locally deployed polyp CADe hardware systems have been commercially available^20^.

Furthermore, the cloud-based workflow may improve accessibility by reducing the need for specialist hardware to be deployed in the hospital, as low-cost computers could be used to access the cloud. This could facilitate new types of procurement, such as per procedure or subscription models, instead of traditional capital equipment purchase. The increased flexibility may open up new ways for hospitals to adopt AI and increase the opportunity to democratize healthcare through better access to the technology.

The use of cloud opens the door to continuously improving the sustainability of AI endoscopy in the years ahead. A traditional AI system deployed on specialist hardware in the hospital will have a fixed energy demand to run the AI model, and any updates would require travel by an engineer to a hospital. This makes it challenging to improve sustainability. Updating 10,000 hospitals would require 10,000 journeys with associated carbon footprints. Cloud-enabled endoscopy lays the foundation to optimize sustainability, exploit new advances in resource-efficient technology, and decrease energy consumption. The cloud enables remote updates that eliminate the need for engineers to travel, optimizes processor mix/selection (CPU, GPU, TPU) to reduce energy consumption, allows resources to be scaled and shared, and makes it easy to adopt new hardware technologies as they become available.

In addition to demonstrating the technical feasibility of a cloud-based CADe workflow, the study demonstrated the algorithm’s efficacy and safety in an age-based screening and surveillance (personal history of colorectal neoplasia) population in a large cohort of endoscopists. The CADe algorithm was trained with an enhanced dataset of clinically significant polyps (large polyps and SSLs) and designed not only to detect smaller polyps but also to identify these more clinically significant lesions, which represented approximately 20% of the training dataset. Consistent with previous studies, the co-primary endpoints of superiority in APC and non-inferiority in PPA were achieved. However, unique improvements in polyp detection were observed for large adenomas, large polyps, and SSLs, findings which have rarely been demonstrated in previous RCTs of CADe algorithms.

Significant increases in the detection of adenomas were observed in the CADe-arm, with 33% more adenomas detected (APC), and an absolute 7.3% increase in ADR. Traditionally, the clinical relevance of polyp detection metrics has been assessed by examining their correlation with PCCRC, the definitive marker of colonoscopy quality, which has an estimated rate of 7.5% in Western populations according to a recent meta-analysis^3,5^. ADR is a well-established quality indicator, consistently demonstrating an inverse relationship with PCCRC. Several studies examining ADR increases of a similar magnitude to those observed in this study (from 35.9% to 43.2%) indicate that such improvements are associated with a lower risk of PCCRC^21–23^. However, with this higher baseline performance of endoscopists, the magnitude of reduction in risk is smaller compared to improvements in those with a lower baseline ADR.

APC, in contrast, is a much more recent and therefore less extensively validated metric. Although it may seem intuitive to assume that an absolute increase in APC of 0.2 (from 0.62 to 0.82) translates into a lower PCCRC risk, only limited data exists in evaluating the correlation of APC with PCCRC. A recent Polish study assessed this, but the highest-performing group had an APC ≥ 0.37, limiting its applicability to the higher APC levels demonstrated in this study^24^. A separate analysis from the New Hampshire Colonoscopy Registry demonstrated a modest reduction in the PCCRC hazard ratios when comparing APC values of 0.50–0.70 with ≥0.70 (HR 0.22 vs. 0.19)^5^. There is a clear need for further research to better clarify the relationship between APC and PCCRC, particularly at APC levels above 0.6. APC has, however, more recently emerged as a valuable metric for identifying suboptimal performance among endoscopists who otherwise meet high-quality benchmarks such as ADR. This is highlighted in the recent American Society for Gastrointestinal Endoscopy (ASGE) colonoscopy quality indicator guidelines, which recommend an APC threshold of 0.6, noting that values up to 1.2 are achievable, underscoring the growing consensus that incremental improvements in APC are thought to carry meaningful clinical relevance, albeit with more research required to confirm this^25^.

The increase in adenoma detection observed in this study, both for APC and ADR, is in line with the recent results of a meta-analysis of CADe RCTs. Importantly, the meta-analysis only identified a significant increase in the detection of smaller-sized adenomas (<10 mm), with no significant increase in large adenomas (≥10 mm)^9^. This is further supported by a recent study, which prospectively assessed a second-generation CADe system to detect large flat lesions, with half of the lesions either not detected or only partially detected after colonoscope position or luminal inflation adjustment^26^. These findings highlight that CADe systems may primarily drive the increase in polyp detection metrics through the detection of smaller-sized polyps, particularly diminutive polyps ( ≤ 5 mm), which are of lower malignant potential and remain of unclear clinical significance in terms of reducing PCCRC occurrences^4^.

In contrast, this study observed a significant two-fold increase in the mean detection of large adenomas and large polyps in the CADe-arm compared to the SoC-arm. Increasing the detection of large polyps is much more likely to reduce PCCRC occurrences, given their established high risk for malignant transformation and yet significant miss-rate^4^. A contributory factor to these novel findings is that the CADe algorithm aided the endoscopists in improving the detection of large non-polypoid polyps, which are more difficult to detect than polypoid lesions. This is evidenced by a significantly higher mean detection of large non-polypoid polyps per-colonoscopy of 0·05 in the CADe-arm compared to 0·01 in the SoC-arm (Ratio 3.34), with a much smaller magnitude of difference for large polypoid polyps. Further supporting this notion is the higher proportion of non-polypoid (‘flat’) morphology in the CADe-arm compared to the SoC-arm for large adenomas (24% vs. 16%) and large polyps (37% vs. 27%). These findings are somewhat intuitive as one would expect that endoscopists less commonly ‘miss’ large polypoid polyps than the more subtle ‘flatter’ polyps, which are notoriously more challenging to detect^4^.

Another noteworthy outcome is the increased detection of SSLs, which was demonstrated in the CADe arm. The meta-analysis of CADe RCTs demonstrated no significant increase in the detection of SSLs, despite an established miss-rate of 27% for these lesions^4,27^. A recent large-scale CADe RCT of more than 2000 patients did demonstrate an improvement in the SSL detection rate, with an adjusted odds ratio of 1.46^28^. However, these findings are modest compared to the almost three-fold increase in SSL detection demonstrated in this study. Several other polyp detection metrics were also superior in the CADe arm. This includes neoplastic polyps, with 39% more neoplastic polyps detected (NPPC), neoplastic serrated polyps (NSP-PPC), which include SSLs and traditional serrated adenomas (TSA), where detection improved two-fold, and more than 40% increased detection of all types of polyps (PPC) in the CADe-arm compared to the SoC-arm.

Polyp detection results stratified by center support the positive overall findings, with detailed results provided in Supplementary Table 8. The detection of adenomas (APC & ADR) increased numerically in seven of the eight centers, with no significant differences observed for any center. Regarding neoplastic polyps, NPPC numerically increased across all centers, SSL-PC across seven (out of the eight centers), and NSP-PC across six centers, with one center (center C) demonstrating a significant improvement across all three neoplastic detection metrics. When considering all polyp types (PPC and PDR), seven centers again showed numerical increases, with significant improvements in PDR at one center (center A) and in PPC at two centers (centers A and E). Notably, the centers showing significant improvements in overall polyp detection metrics (A and E) were distinct from the one (center C) with significant improvements in neoplastic detection metrics. There was also no significant increase observed in any polyp detection metrics at center B, which had previously contributed less than 1% of the total CADe developmental dataset.

When considering the adoption of any technology, it is equally important to assess for potential unwanted effects along with its effectiveness. Clinical concerns surrounding CADe systems have primarily been related to their potential to increase unnecessary resections (such as normal mucosa) and CWT. Reassuringly, the CADe system was non-inferior to the SoC-arm for PPA^29,30^. This metric, which evaluates the proportion of resections that were polyps, was numerically higher in the CADe arm. Both CWT and procedure time were significantly higher in the CADe-arm, although the increase was only by 1 min. Given that CWT included polypectomy time, and more polyps were detected in the CADe-arm, this increase likely reflects improved polyp detection rather than a negative outcome. The average procedure time for negative procedures, where no polyps were detected, was 8 min in both arms, suggesting that the CADe system does not unnecessarily prolong colonoscopy procedure time.

The strengths of this study include its multi-center, multi-nation design and the large cohort of endoscopists (more than 20) evaluated. The study was also performed in an average-risk population, and analysis limited to colonoscopies of adequate procedural quality in line with societal guidelines such as adequate bowel preparation, cecal intubation and CWT above 6 min, which is likely to reduce biases between the two study arms.

The study also contains limitations. The study did not include endoscopists with a lower ADR ( < 25% ADR). Patient recruitment was limited to age-based screening and surveillance indications for colonoscopy; the impact of CADe was not evaluated on symptomatic or high-risk (e.g., FIT-positive) indications for colonoscopy. At the time of the study, the CADDIE system’s indications-for-use limited it to only Olympus endoscopic equipment. The study did not contain any other manufacturer’s equipment, and no analysis has been performed on the study data to evaluate the generalizability of the algorithm’s performance beyond the Olympus equipment used in the study. The primary endpoints of the study are limited to adenoma detection and safety. The study does not examine longer-term patient outcomes, such as the impact of CADe on PCCRC. Further research, including real-world evidence generation, would help to evaluate the effectiveness, safety, utilization, and outcomes of the CADe algorithm outside of the RCT setting.

In summary, the emergence of real-time cloud AI endoscopy represents a paradigm shift in the way gastroenterology departments can access computer-aided systems. It has the potential to power a new generation of AI systems for the detection, diagnosis, and treatment of diseases, where doctors can seamlessly access the latest AI models and new products targeting new disease areas, without being limited by legacy hardware or in-person maintenance from engineers. This paper presented a multi-country, multi-center RCT demonstrating the feasibility, efficacy, and safety of a real-time cloud-based polyp CADe system in endoscopy. The centers in the study had no specialist IT equipment or infrastructure, indicating the potential for adoption of real-time cloud endoscopy in standard hospitals and routine clinical practice. Furthermore, the study observed an increase in endoscopists’ detection of polyps across several detection metrics when using CADe, most notably for large adenomas, large polyps, and sessile serrated lesions. These findings indicate that CADe systems may contribute to ongoing efforts aimed at improving patient outcomes. Future research directions include developing new AI training methods to further enhance the detection of specific lesion types and exploring the impact of optimizing training dataset enrichment on computer-aided systems performance for CADe and beyond. Future work also may include evaluating the generalizability of the algorithm and effectiveness in a wider clinical setting (e.g., symptomatic patients, lower ADR endoscopists).

## Methods

### CADDIE system

CADDIE (Odin Medical Ltd., London, UK) is the first CE-Marked/MDR-certified cloud-based AI software for real-time polyp detection during colonoscopy, enabling remote software updates of the algorithm. The CADe algorithm used throughout the EAGLE Trial was fixed at version 1.4. The CADe convolutional neural network (CNN) algorithm was developed with over 1000 histologically confirmed polyps, including an enhanced subset of clinically significant polyps, with large polyps and SSLs collectively making up approximately 20% of the training data. Large polyps accounted for more than 10%, exceeding their 5.0–7.0% prevalence in clinical practice, while SSLs also comprised over 10%, surpassing the 4.6% prevalence reported in a recent EU-US meta-analysis^4,31^. The training data includes hyperplastic polyps and SSLs that have undergone expert pathologist diagnosis.

**Fig. 3:**
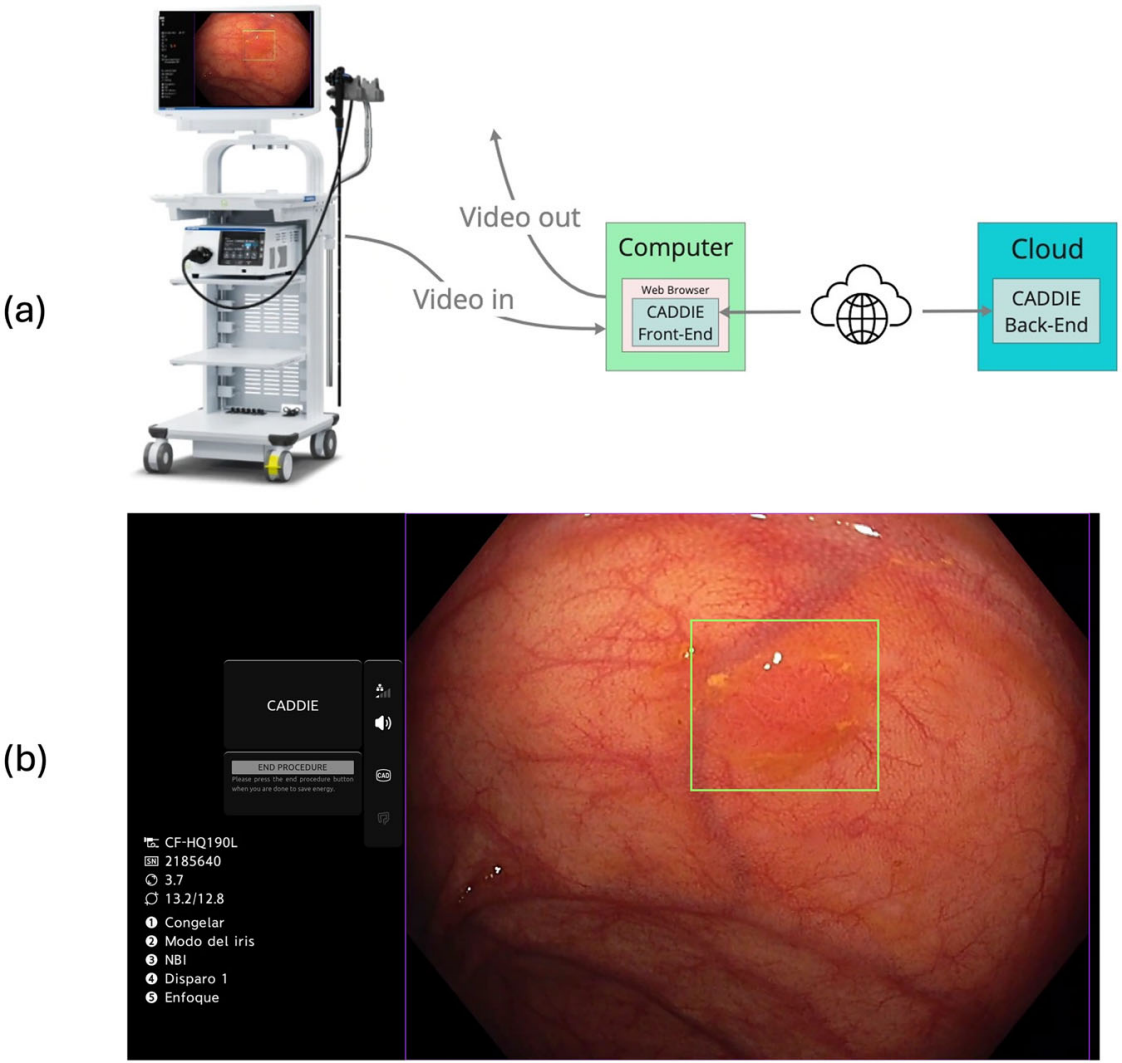
Real-time cloud-based CADDIE system*.* **a** The CADDIE polyp detection (CADe) algorithm is deployed on a cloud. It is accessed using a secure web browser on a computer connected to the endoscopy processor and monitor. The cloud-based algorithm receives the video data via the hospital internet network, analyses it, and returns a green bounding box overlay (if appropriate) to the endoscopy monitor in real-time to highlight the suggested presence and location of a polyp. **b** Demonstration of the visible on-screen purpose bounding box which delineates the active image capture zone that is sent to the cloud for AI inference. Data outside of this zone is not sent to the cloud. Images provided with permission by Olympus and **a** created using Concept map (Miro visual workspace [2025]).

CADDIE is a web-based, cloud-integrated CADe system with a front-end client in the endoscopy suite and a cloud-based back-end. CADDIE integrates with standard endoscopy equipment (Fig. 3a) via a Chromium-based web browser, connected to the endoscopy video processor through a frame capture card and SDI connection for real-time acquisition of the colonoscopy video feed from the endoscopy image processor (EIP). The video stream is simultaneously displayed on the endoscopy screen and transmitted via a secure connection to the cloud-based AI processing pipeline over the internet.

**Fig. 4:**
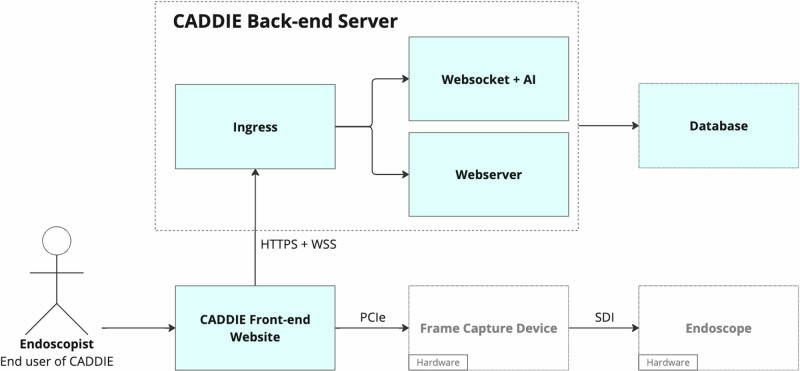
The cloud-deployed CADDIE webapp architecture. The software components are displayed in light blue. The endoscopist operates the device through the front-end website. The website is accessed on the client computer, which has a PCIe frame capture card, receiving the video feed through an SDI connector from the endoscope image processor. The front-end website transmits the video frames to the back-end, where an ingress forwards these to the websocket for AI processing. The webserver component serves the website over HTTPS and reads/writes the user configuration to a database. Images provided with permission by Olympus and created using Concept map (Miro visual workspace [2025]).

The cloud back-end applies the CADe algorithm, streaming detection results back to the endoscopy suite, in real-time, as bounding boxes overlaid on the video feed on the endoscopy screen (Fig. 4). CADDIE is cloud-agnostic, deployable across multiple cloud providers and compliant with international data security standards (ISO 27001, HIPAA, SOC2, GDPR). Its microservice-based architecture enables modular deployment and system improvements without disrupting clinical workflows.

To maintain performance, CADDIE requires minimal cloud network latency for real-time polyp detection. This includes the time to transmit the video to the cloud and the return of the bounding box/marker annotation to the hospital for visualization. A latency below 100 ms is required by the manufacturer. This requirement is related to marker annotation delay in other traditional CADe systems^32^. To ensure uninterrupted real-time operation, a minimum internet connection of 8 megabits per second (Mbps) upstream and 1Mbps downstream is required. This was validated prior to the installation of the equipment at each hospital in the study. A wired Ethernet connection was used for network stability, while the low-bandwidth communication technology enabled ultra-low latency processing, ensuring efficient real-time detection. As a risk mitigation measure, when the cloud network latency surpasses a predefined threshold, the system automatically disables the display of the bounding-box overlay and alerts the user of this.

From a security perspective, CADDIE complies with General Data Protection Regulation (GDPR), the UK Data Protection Act, and the Health Insurance Portability and Accountability Act (HIPAA), ensuring no personally identifiable information (PII) or protected health information (PHI) is processed, stored, or transmitted. Only de-identified image data is sent to the cloud for AI inference, with site-specific configurations ensuring PII is excluded. A purple bounding box delineates the active image capture zone, preventing unintentional PII transmission (Fig. 3b). End users can verify this configuration at any time to maintain compliance. Data transmission is encrypted end-to-end between the endoscopy suite and the cloud using Transport Layer Security (TLS 1.2+). Ephemeral data is encrypted at rest using FIPS 140-2 compliant modules, providing further protection against unauthorized access.

### Study design

The EAGLE study is a prospective, multi-center, parallel-group RCT conducted in eight European centers: Italy (*n* = 3), Poland (*n* = 3), Germany (*n* = 1) and Spain (*n* = 1) (Supplementary Table 1). The study was developed to support an FDA 510(k) submission and incorporated regulatory feedback and guidance. The CADDIE medical device was FDA cleared in July 2024. Ethical approval was obtained independently at all sites before recruitment. The study was registered on ClinicalTrials.gov (NCT05730192) on 15th February 2023 and reported according to CONSORT-AI extension^33^.

### Patients

Patients aged ≥40 years, scheduled for screening or surveillance colonoscopy (at least 3 years after the last examination) were eligible for inclusion. To align with the US average-risk CRC population, exclusion criteria included high-risk indications of colonoscopy (e.g., polyposis syndromes, previous CRC, and FIT-positive), inflammatory bowel disease (IBD), previous colonic resection, emergency colonoscopy, planned elective therapeutic colonoscopy and biopsy/polypectomy contraindications. Eligible patients with a negative FIT test were not excluded. All eligible patients provided written consent before recruitment. Participating endoscopists were required to have a baseline of at least 1000 colonoscopy procedures and an adenoma detection rate (ADR) of ≥25%, per US guidelines^25^.

### Randomization and masking

Recruiting research staff at each site, randomized patients immediately before colonoscopy, assigning patients in a 1:1 ratio in blocks of four to CADDIE-assisted colonoscopy (CADe-arm) or standard colonoscopy (SoC-arm). Block randomization was conducted at the study level (i.e., not site level), ensuring allocation concealment. Patient enrollment and group allocation were managed via an online electronic data capture (EDC) system (Catchtrial). The research nurse and endoscopist were unmasked just before the procedure. Blinding the endoscopist was unfeasible due to CADDIE’s interactive nature. However, study endpoints relied on histopathological diagnoses by a blinded pathologist. Furthermore, the trial statistician (Statsconsultancy Ltd) received study data only at the end of the study, maintaining analytical blinding.

### Procedures

SoC-arm patients underwent routine colonoscopy per standard of care (i.e., without the CADe system). In the CADe-arm, CADDIE was enabled for at least withdrawal from the cecum, with insertion at the endoscopist’s discretion. Endoscopists had no prior experience with CADDIE, and its use was limited to the trial. The Sponsor trained all investigators and study staff on CADDIE’s user interface and intended use. During the trial, each endoscopist was required to complete a minimum of ten colonoscopies from recruited patients.

Colonoscopies were conducted using high-definition monitors with white-light imaging and EVIS X1 endoscopy system. To comply with regulatory requirements, the study protocol limited endoscopes to Olympus models that were already FDA-cleared, or in the process of being cleared by the FDA, and expected to receive FDA clearance and be available on the US market before study completion. This includes the following Olympus endoscopes:CF-HQ190L/I, CF-H190L/I, PCF-H190L/I, PCF-H190TL/I, PCF-PH190L/I, PCF-HQ190L/I, CF-HQ1100DL/I and CF-EZ1500DI. In line with these requirements, procedures performed with CF-EZ1500DI endoscopes were subsequently excluded because this model was not FDA-cleared by the end of the study.

Sedation use was at the endoscopist’s discretion. Bowel preparation was assessed via the Boston Bowel Preparation Score (BBPS) with inadequate procedures excluded (BBPS < 2 in any colonic segment, failed cecal intubation, or colonoscopy withdrawal time (CWT) below 6 min) to reduce bias, as these factors correlate with polyp detection^25^.

Each polyp was measured using an instrument of known size, and morphology classified using the Paris classification. Polyps were placed in separate specimen pots, with histopathology reported per WHO classification. Patients were followed up within 24 h post-procedure to document adverse events.

### Clinical outcome measures

The study assessed two co-primary endpoints. The primary efficacy outcome was the mean Adenomas Per Colonoscopy (APC), defined as the proportion of adenomas (tubular, villous, and tubulovillous adenoma) and adenocarcinomas per colonoscopy. To assess CADDIE’s impact on unnecessary resections, the co-primary safety endpoint was Percent Positive Agreement (PPA), defined as the percentage of resections histologically confirmed as adenomas, SSLs, or proximal large ( ≥ 10 mm) hyperplastic polyps.

Secondary outcomes included adenoma detection-rate (ADR), mean neoplastic serrated polyps per colonoscopy (NSP-PPC), and mean neoplastic polyps per-colonoscopy (NPPC), compared between study arms. Exploratory outcomes included polyp detection-rate (PDR), mean polyps per-colonoscopy (PPC), and mean SSLs per colonoscopy (SSL-PC). Definitions of these metrics are outlined in Supplementary Table 2.

Sub-group analyses for APC and PPC comparisons were based on size (diminutive [≤5 mm], small [6–9 mm], large [≥10 mm]), location (proximal vs. distal), and morphology (polypoid vs. non-polypoid). The proximal colon included the cecum, ascending colon, hepatic flexure, and transverse colon; the rest was distal. Polypoid polyps were Paris classification Ip, Isp and Is; others were non-polypoid.

Secondary procedural outcomes included CWT and total procedural time. Adverse events (AE) were assessed by severity and relation to the intervention.

### Technical outcome measures

CADDIE’s real-time detection performance during the study was investigated using an exploratory analysis of the cloud network latency. This metric is dependent on the hospital network and connection to the cloud. It was analyzed across the duration of all study procedures, with a subgroup analysis by site.

### Statistical analysis

The co-primary outcomes were APC and PPA. The sample size for both co-primary endpoints was calculated. The difference in APC between groups was assessed on a superiority basis. The study was powered to detect an APC increase from 0.83 to 1.02, with the mean value in the control arm estimated to be 0.83^34^. The standard deviation of the APC values was assumed to be 0.993. Using a one-sided 2.5% significance level (equivalent to a two-sided 5% significance level) and 80% power, it was calculated that 430 patients per group were required, totaling 860 patients. PPA was powered for non-inferiority. The PPA in the control arm was estimated to be 75.7% (based on the literature)^35^. A non-inferiority margin of 15% non-inferiority was assumed. Using a one-sided 2.5% significance level and 80% power, it was calculated that 129 resections per arm were required (258 in total). To satisfy both co-primary endpoints (APC and PPA), the sample size was selected as the highest of the two individual calculations, which was APC (*n* = 860). This sample size resulted in an increased effective power for PPA of 99.92%, and a combined study power of 80%. Accounting for a 10% dropout rate, the planned sample size was 946 patients.

The study was analyzed using the modified intention to treat (mITT) population per-protocol, and in line with the FDA-cleared predicate device and other 510(k) cleared devices. Exclusions are outlined in the results section. Due to a higher than anticipated dropout rate, it was permitted during the study to revise the planned sample size recruitment up to 985 patients (an additional 39 patients).

Categorical data were presented as frequency counts and percentages, while continuous data were reported as means and SDs. Baseline demographics, clinical characteristics, and colonoscopy quality parameters were compared using Chi-square tests (categorical variables) and two-sample *t*-tests (continuous variables). Generalized Linear Mixed Models (GLMM) were used to compare endpoints and subgroups. The primary efficacy endpoint (APC) was evaluated with a mixed Generalized Linear Model (GLM) for positively skewed distributions (negative binomial with log link function), using a two-sided 5% significance level. PPA non-inferiority was assessed using a mixed GLM (binomial distribution with identity link function). No statistical difference was observed between study arms regarding patients’ demographics, colonoscopy indication (Supplementary Table 3), or study center (Supplementary Table 1). Therefore, the final statistical models included study arm as a fixed effect and endoscopist as a random effect.

Secondary and exploratory mean polyp detection metrics were analyzed using the same methods as APC, while ADR and PDR followed the PPA methodology. Analysis of polyp detection metrics stratified by center followed this methodology, except in cases where an outcome occurred in one group but not the other. In such instances, negative binomial regression could not be applied, and a Mann–Whitney test was used instead. CWT and total procedure times were analyzed using mixed linear regression. Group differences were expressed as a ratio with 95% confidence interval (CI), except PPA, which used a one-sided 97.5% CI. A *p* value ≤ 0.05 was considered statistically significant. Data analysis was conducted by Statsconsultancy Ltd (Amersham, UK) using Stata (version 15.1).

### Role of the funding source

The study funder and sponsor, Odin Medical Ltd., provided the CADe system and assisted in the study design. Meditrial Europe, an independent clinical research organization, conducted the study, and Statsconsultancy Ltd, an independent statistical organization, performed the statistical analysis.

### Patient and public involvement

Patient and public involvement was not involved in the design of the study.

## Supplementary information

Supplementary Material

## Data Availability

The datasets generated and/or analyzed during the current study will be made available to researchers upon reasonable request and dependent on the necessary relevant approvals.
