# Supplementary material for: A novel cloud-based artificial intelligence for real-time detection of colorectal neoplasia – a randomized controlled trial (EAGLE)
Source: NPJ Digit Med. 2025 Dec 26;9:84. doi: 10.1038/s41746-025-02270-1 (PMC12852673; doi:10.1038/s41746-025-02270-1)
Supplement: Supplementary file 1 — Supplementary Material [file 41746_2025_2270_MOESM1_ESM.pdf]

Supplementary Material

Supplementary Table 1: Total recruitment for each site……………………………………….2
Supplementary Table 2: Definition of polyp detection metrics and withdrawal time………3
Supplementary Table 3: Patient characteristics and procedural indications……….………4
Supplementary Table 4: Distribution of endoscope models across the two arms, AI-assisted (CADe-Arm) or standard of care (SoC-Arm) colonoscopy…………………………………...5
Supplementary Table 5: Procedural characteristics…………………………………………6
Supplementary Table 6: Cloud network latency results for each center and overall across the study ………………………………...…………………………………………………...7
Supplementary Table 7: Polypoid and non-polypoid APC and PPC results for overall and large sized polyps…………...……………………………………………………………8
Supplementary Table 8 (a-g): Polyp detection metrics result stratified by center…..………9
CONSORT-AI Extension……………………..……………………………………….16

<table>
<tr><th rowspan="2">Center</th><th colspan="2">All patients
[N=841]</th><th colspan="2">SoC [N=424]</th><th colspan="2">CADe
[N=417]</th><th rowspan="2">P-value</th></tr>
<tr><th>N</th><th>Summary</th><th>N</th><th>Summary</th><th>N</th><th>Summary</th></tr>
<tr><td>Center A</td><td rowspan="8">841</td><td>35 (4.2%)</td><td rowspan="8">424</td><td>18 (4.3%)</td><td rowspan="8">417</td><td>17 (4.1%)</td><td rowspan="8">0.97</td></tr>
<tr><td>Center B</td><td>151 (18.0%)</td><td>74 (17.5%)</td><td>77 (18.5%)</td></tr>
<tr><td>Center C</td><td>152 (18.1%)</td><td>79 (18.6%)</td><td>73 (17.5%)</td></tr>
<tr><td>Center D</td><td>98 (11.7%)</td><td>53 (12.5%)</td><td>45 (10.8%)</td></tr>
<tr><td>Center E</td><td>152 (18.1%)</td><td>77 (18.2%)</td><td>75 (18.0%)</td></tr>
<tr><td>Center F</td><td>91 (10.8%)</td><td>47 (11.1%)</td><td>44 (10.6%)</td></tr>
<tr><td>Center G</td><td>45 (5.4%)</td><td>20 (4.7%)</td><td>25 (6.0%)</td></tr>
<tr><td>Center H</td><td>117 (13.9%)</td><td>56 (13.2%)</td><td>61 (14.6%)</td></tr>
</table>

Supplementary Table 1: Total recruitment for each site. Summary statistics are presented as numbers (percentage). Several years prior to the study, Center B had previously contributed a small number of cases to the developmental dataset (<1%) from a different location in the hospital using different equipment.

**Adenoma Detection Rate (ADR)**	The percentage of procedures with histologically confirmed adenomas (tubular adenoma, villous, tubulovillous) and adenocarcinomas divided by total number of procedures performed.
**Adenomas Per Colonoscopy (APC)**	The total number of histologically confirmed adenomas and adenocarcinomas divided by the total number of procedures performed
**Colonoscope withdrawal time**	The time it takes to withdraw the colonoscope from the furthest point (TI or cecum) back to the rectum.
**Colonoscope withdrawal time of negative procedures only**	The time it takes to withdraw the colonoscope from the furthest point (TI or caecum) for colonoscopies that did not require any polyp biopsies or resections.
**Neoplastic Polyps Per Colonoscopy (NPPC)**	The total number of histologically confirmed adenomas, adenocarcinomas, sessile serrated lesions, and traditional serrated adenomas divided by the total number of procedures performed.
**Neoplastic Serrated Polyps Per Colonoscopy (NSP-PC)**	The total number of histologically confirmed sessile serrated lesions, serrated lesion with cytological dysplasia, or traditional serrated adenoma, divided by the total number of procedures performed
**Positive Percent Agreement (PPA)**	The percent of histologically confirmed adenomas, sessile serrated adenomas and large (>10mm) hyperplastic polyps of the proximal colon (cecum, ascending colon, hepatic flexure, and transverse colon) divided by the total number of resections.
**Polyp Detection Rate (PDR)**	The total number of histologically confirmed adenomas, adenocarcinomas, hyperplastic, sessile serrated lesions, and traditional serrated adenomas divided by the total number of procedures performed.
**Polyps Per Colonoscopy (PPC)**	The total number of histologically confirmed polyps detected (adenomas, adenocarcinomas, sessile serrated adenoma, traditional serrated adenoma, hyperplastic), divided by the total number of colonoscopies.
**SSLs Per Colonoscopy (SSL-PC)**	The total number of histologically confirmed sessile serrated lesions divided by the total number of procedures performed

Supplementary Table 2: Definition of polyp detection metrics and withdrawal time.

Variable	Category	All patients [N=841]		SoC [N=424]		CADe [N=417]		P-value
		N	Summary	N	Summary	N	Summary	
**Age**	-	841	58·5 ± 9·3	424	58·2 ± 9·5	417	58·7 ± 9·0	0·43
**Gender**	Female	841	412 (49·0%)	424	218 (51·4%)	417	194 (46·5%)	0·21
	Male		428 (50·9%)		205 (48·4%)		223 (53·5%)	
	Other		1 (0·1%)		1 (0·2%)		0 (0·0%)	
**Ethnicity**	Non-Latino/Hispanic	827	686 (82·9%)	415	349 (84·1%)	412	337 (81·8%)	0·38
	Latino/Hispanic		141 (17·1%)		66 (15·9%)		75 (18·2%)	
**Race**	Caucasian	840	837 (99·6%)	424	423 (99·8%)	416	414 (99·5%)	0·25
	African American		1 (0·1%)		1 (0·2%)		0 (0·0%)	
	Other		2 (0·2%)		0 (0·0%)		2 (0·5%)	
**ASA score**	I	841	635 (75·5%)	424	321 (75·7%)	417	314 (75·3%)	0·76
	II		198 (23·5%)		100 (23·6%)		98 (23·5%)	
	III		8 (1·0%)		3 (0·7%)		5 (1·2%)	
**Colonoscopy Indication**	Screening	841	653 (77·7%)	424	329 (77·6%)	417	324 (77·7%)	0·97
	Surveillance		188 (22·3%)		95 (22·4%)		93 (22·3%)	

Supplementary Table 3: Patient characteristics and procedural indications. Summary statistics are mean ± standard deviation, or number (percentage). Missing data included ethnicity for 14 patients, and race for 1 patient.

Olympus Endoscope Model	CADe-Arm	SoC-Arm	Grand Total
CF-H190L/I	52	60	112
CF-HQ1100DL/I	23	26	49
CF-HQ190L/I	316	319	635
CF-HQ 1100DI	10	8	18
PCF-H190L/I	16	11	27
**Grand Total**	**417**	**424**	**841**

Supplementary Table 4: Distribution of endoscope models across the two arms, AI-assisted (CADe-Arm) or standard of care (SoC-Arm) colonoscopy.

Characteristic	Outcome	Study Arm	N patients	Summary (*)	Difference (#) (95% CI)	P-value
**Sedation**	**Sedation (None)**	SoC	424	29 (6·8%)	1 ±±	0·70
		CADe	417	29 (7·0%)		
	**Sedation (Conscious)**	SoC	424	150 (35·4%)	0·91 (0·68, 1·21) ±±	
		CADe	417	139 (33·3%)		
	**Sedation (Deep)**	SoC	424	245 (57·8%)	1·08 (0·82, 1·43) ±±	
		CADe	417	249 (59·7%)		
**Bowel Preparation**	**BBPS total**	SoC	424	8 [6, 9]	0	0·81
		CADe	417	8 [6, 9]		
**Withdrawal time**	**Withdrawal time (neg)** (+)	SoC	178	8 [7, 10]	1	0·44
		CADe	138	8 [7, 11]	1·02 (0·96, 1·09)	
	**Withdrawal time (all)** (++)	SoC	424	10 [8, 13]	1	**<0·001**
		CADe	417	11 [8, 15]	1·10 (1·05, 1·15)	
**Procedure time**	**Procedure time (neg)** (+)	SoC	178	17 [13, 21]	1	0·73
		CADe	138	17 [14, 20]	1·01 (0·94, 1·09)	
	**Procedure time (all)** (++)	SoC	424	18 [15, 24]	1	**0·003**
		CADe	417	19 [16, 26]	1·07 (1·02, 1·12)	

Supplementary Table 5: Procedural characteristics.
BBPS = Boston Bowel Preparation Score
(*) Summary statistics are number (percentage) or median [inter-quartile range]
(#) Ratio of values in CADe relative to SoC
(+) Analysis performed only for 'negative' procedures with no polyps found
(++) Analysis for all procedures
(±±) Odds Ratios represent the odds of being in either conscious/deep sedation categories (relative to the no sedation category) for the CADe group relative to the SoC group

Center	Total time (m)	Latency > 100ms (m)	% Exceeding 100ms
**A**	1043	0·9	0·08
**B**	5709	8·3	0·15
**C**	7250	23	0·32
**D**	3674	43	1·17
**E**	4906	38·2	0·78
**F**	3633	0	0
**G**	1028	0	0
**H**	3908	1·4	0·04
**Overall**	**31151**	**114·8**	**0·37**

Supplementary Table 6: Cloud network latency results for each center and overall across the study. M = minutes, ms = milliseconds.

Polyp Metric	Size	Outcome	Study Arm	n patients	Total	Mean ± SD	Difference Ratio (95% CI)	P-value
Adenomas (APC)	Overall	**Polypoid**	SoC	424	160	0.38 ± 0.94	1	**0.02**
			CADe	417	211	0.51 ± 0.96	1.39 (1.06, 1.83)	
		**Non-polypoid**	SoC	424	75	0.18 ± 0.62	1	**0.04**
			CADe	417	108	0.26 ± 0.91	1.57 (1.01, 2.44)	
	Large (≥10 mm)	**Polypoid**	SoC	424	16	0.04 ± 0.20	1	0.12
			CADe	417	27	0.06 ± 0.29	1.71 (0.87, 3.35)	
		**Non-polypoid**	SoC	424	3	0.01 ± 0.11	1	0.29
			CADe	417	8	0.02 ± 0.22	2.71 (0.43, 16.9)	
Polyps (PPC)	Overall	**Polypoid**	SoC	424	242	0.57 ± 1.11	1	**0.02**
			CADe	417	308	0.74 ± 1.31	1.33 (1.06, 1.69)	
		**Non-polypoid**	SoC	424	168	0.40 ± 1.00	1	**0.03**
			CADe	417	221	0.53 ± 1.29	1.37 (1.02, 1.84)	
	Large (≥10 mm)	**Polypoid**	SoC	424	16	0.04 ± 0.20	1	**0.04**
			CADe	417	31	0.07 ± 0.30	1.95 (1.02, 3.70)	
		**Non-polypoid**	SoC	424	6	0.01 ± 0.14	1	**0.03**
			CADe	417	19	0.05 ± 0.29	3.34 (1.10, 10.2)	

Supplementary Table 7: Polypoid and non-polypoid APC and PPC results for overall and large sized polyps. Group differences are ratio of values in CADe relative to SoC. Note that 132 of the total 1222 lesions have missing morphology data and were excluded from the analysis.

Metric	Study Arm	N patients	Total	Mean ± SD	Difference Ratio (95% CI)	P-value
A	SoC	18	8	0·44 ± 0·78	1	0·74
	CADe	17	9	0·53 ± 0·72	1·19 (0·43, 3.32)	
B	SoC	74	63	0·85 ± 1·35	1	0·41
	CADe	77	79	1.03 ± 1·43	1·22 (0·77, 1·93)	
C	SoC	79	49	0·62 ± 1·50	1	0·08
	CADe	73	77	1·05 ± 1·87	1·70 (0·95, 3·05)	
D	SoC	53	26	0·49 ± 1·34	1	0·34
	CADe	45	30	0·67 ± 1·07	1·48 (0·67, 3·28)	
E	SoC	77	28	0·36 ± 0·72	1	0·28
	CADe	75	37	0·49 ± 0·84	1·36 (0·78, 2·39)	
F	SoC	47	23	0·49 ± 0·66	1	0·40
	CADe	44	28	0·64 ± 0·99	1·30 (0·71, 2·39)	
G	SoC	20	12	0·60 ± 1·10	1	1·00
	CADe	25	15	0·60 ± 0·76	1·00 (0·42, 2·38)	
H	SoC	56	53	0·95 ± 1·27	1	0·57
	CADe	61	68	1·11 ± 1·91	1·18 (0·67, 2·08)	

Supplementary Table 8 (a): Adenomas per colonoscopy (APC) results stratified by center.

Metric	Study Arm	N patients	n (%)	Difference Ratio (95% CI)	P-value
A	SoC	18	5 (27.8%)	0	0.40
	CADe	17	7 (41.2%)	13.4% (-17.8%, 44.6%)	
B	SoC	74	35 (47.3%)	0	0.80
	CADe	77	38 (49.4%)	2.1% (-13.9%, 18.0%)	
C	SoC	79	25 (31.7%)	0	0.08
	CADe	73	33 (45.2%)	13.6% (-1.8%, 28.9%)	
D	SoC	53	14 (26.4%)	0	0.23
	CADe	45	17 (37.8%)	11.4% (-7.1%, 29.8%)	
E	SoC	77	21 (27.3%)	0	0.25
	CADe	75	27 (36.0%)	8.7% (-6.0%, 23.5%)	
F	SoC	47	19 (40.4%)	0	0.86
	CADe	44	17 (38.6%)	-1.8% (-21.9%, 18.3%)	
G	SoC	20	7 (35.0%)	0	0.37
	CADe	25	12 (48.0%)	13.0% (-15.6%, 41.6%)	
H	SoC	56	26 (46.4%)	0	0.90
	CADe	61	29 (47.5%)	1.1% (-17.0%, 19.2%)	

Supplementary Table 8 (b): Adenomas detection rate (ADR) results stratified by center.

Metric	Study Arm	N patients	Total	Mean ± SD	Difference Ratio (95% CI)	P-value
A	SoC	18	8	0·44 ± 0·78	1	0·22
	CADe	17	14	0·82 ± 1·07	1·85 (0·68, 5·01)	
B	SoC	74	63	0·85 ± 1·35	1	0·36
	CADe	77	81	1·05 ± 1·45	1·24 (0·78, 1·97)	
C	SoC	79	50	0·63 ± 1·50	1	**0·02**
	CADe	73	89	1·22 ± 1·90	1·92 (1·13, 3·30)	
D	SoC	53	29	0·55 ± 1·35	1	0·25
	CADe	45	34	0·76 ± 1·11	1·54 (0·74, 3·22)	
E	SoC	77	29	0·38 ± 0·73	1	0·33
	CADe	75	37	0·49 ± 0·84	1·32 (0·76, 2·28)	
F	SoC	47	28	0·60 ± 0·74	1	0·34
	CADe	44	34	0·77 ± 1·01	1·30 (0·76, 2·21)	
G	SoC	20	17	0·85 ± 1·09	1	0·85
	CADe	25	23	0·92 ± 1·66	1·08 (0·48, 2·45)	
H	SoC	56	56	1·00 ± 1·26	1	0·66
	CADe	61	69	1·13 ± 1·98	1·13 (0·65, 1·96)	

Supplementary Table 8 (c): Neoplastic polyps per colonoscopy (NPPC) results stratified by center.

Metric	Study Arm	N patients	Total	Mean ± SD	Difference Ratio (95% CI)	P-value
A	SoC	18	0	0·00 ± 0·00	-	0·46
	CADe	17	5	0·29 ± 0·99	-	
B	SoC	74	0	0·00 ± 0·00	-	1·00
	CADe	77	2	0.03 ± 0·23	-	
C	SoC	79	1	0·01 ± 0·11	1	**0·02**
	CADe	73	12	0·16 ± 0·44	13.0 (1·63, 103)	
D	SoC	53	3	0·06 ± 0·23	1	0·60
	CADe	45	4	0·09 ± 0·36	1·57 (0·30, 8·35)	
E	SoC	77	1	0·01 ± 0·11	-	1·00
	CADe	75	0	0·00 ± 0·00	-	
F	SoC	47	5	0·11 ± 0·31	1	0·69
	CADe	44	6	0·14 ± 0·41	1·28 (0·38, 4·36)	
G	SoC	20	5	0·25 ± 0·44	1	0·87
	CADe	25	8	0·32 ± 1·25	1·16 (0·19, 7·03)	
H	SoC	56	3	0·05 ± 0·30	1	0·39
	CADe	61	1	0·02 ± 0·13	0·31 (0·02, 4·49)	

Supplementary Table 8 (d): Neoplastic serrated polyps per colonoscopy (NSP-PC) results stratified by center. (-) = Mann–Whitney test used for analysis as an outcome occurred in one group but not the other, therefore difference ratio not calculated.

Metric	Study Arm	N patients	Total	Mean ± SD	Difference Ratio (95% CI)	P-value
A	SoC	18	0	0·00 ± 0·00	-	0·46
	CADe	17	5	0·29 ± 0·99	-	
B	SoC	74	0	0·00 ± 0·00	-	1·00
	CADe	77	2	0.03 ± 0·23	-	
C	SoC	79	0	0·00 ± 0·00	-	**0·01**
	CADe	73	8	0·11 ± 0·36	-	
D	SoC	53	3	0·06 ± 0·23	1	0·60
	CADe	45	4	0·09 ± 0·36	1·57 (0·30, 8·35)	
E	SoC	77	1	0·01 ± 0·11	-	1·00
	CADe	75	0	0·00 ± 0·00	-	
F	SoC	47	4	0·09 ± 0·28	1	0·48
	CADe	44	6	0·14 ± 0·41	1·60 (0·43, 6·00)	
G	SoC	20	3	0·15 ± 0·37	1	0·52
	CADe	25	8	0·32 ± 1·25	2·13 (0·21, 21·9)	
H	SoC	56	0	0·00 ± 0·00	-	1·00
	CADe	61	1	0·02 ± 0·13	-	

Supplementary Table 8 (e): Sessile serrated lesions per colonoscopy (SSL-PC) results stratified by center. (-) = Mann–Whitney test used for analysis as an outcome occurred in one group but not the other, therefore difference ratio not calculated.

Metric	Study Arm	N patients	Total	Mean ± SD	Difference Ratio (95% CI)	P-value
A	SoC	18	9	0·50 ± 0·79	1	**0·04**
	CADe	17	20	1·18 ± 1·24	2·35 (1·02, 5.43)	
B	SoC	74	93	1·26 ± 1·61	1	0·16
	CADe	77	130	1·68 ± 2·09	1·34 (0·89, 2·02)	
C	SoC	79	75	0·95 ± 1·78	1	0·07
	CADe	73	106	1·45 ± 2·19	1·53 (0·96, 2·43)	
D	SoC	53	42	0·79 ± 1·57	1	0·61
	CADe	45	41	0·91 ± 1·22	1·18 (0·62, 2·27)	
E	SoC	77	44	0·57 ± 0·99	1	**0·03**
	CADe	75	71	0·95 ± 1·43	1·72 (1·04, 2·83)	
F	SoC	47	47	1·00 ± 1·00	1	0·08
	CADe	44	63	1·43 ± 1·37	1·43 (0·95, 2·15)	
G	SoC	20	30	1·50 ± 1·19	1	0·57
	CADe	25	31	1·24 ± 1·71	0·84 (0·46, 1·52)	
H	SoC	56	111	1·98 ± 1·85	1	0·79
	CADe	61	127	2·08 ± 2·20	1·05 (0·73, 1·51)	

Supplementary Table 8 (f): Polyps per colonoscopy (PPC) results stratified by center.

Metric	Study Arm	N patients	Polyp detected n (%)	Difference % (95% CI)	P-value
A	SoC	18	6 (33·3%)	0	**0·02**
	CADe	17	12 (70·6%)	37·3 (6·5, 68·0)	
B	SoC	74	42 (56·8%)	0	0·59
	CADe	77	47 (61·0%)	4·3 (-11·4, 20·0)	
C	SoC	79	38 (48·1%)	0	0·18
	CADe	73	43 (58·9%)	10·8 (-5·0, 26·6)	
D	SoC	53	23 (43·4%)	0	0·75
	CADe	45	21 (46·7%)	3·2 (-16·5, 23·0)	
E	SoC	77	29 (37·7%)	0	0·25
	CADe	75	35 (46·7%)	8·9 (-6·4, 24·2)	
F	SoC	47	30 (63·8%)	0	0·36
	CADe	44	32 (72·7%)	8·9 (-10·1, 27·9)	
G	SoC	20	16 (80·0%)	0	0·13
	CADe	25	15 (60·0%)	-20·0 (-46·0, 6·0)	
H	SoC	56	41 (73·2%)	0	0·79
	CADe	61	46 (75·4%)	2·2 (-13·7, 18·0)	

Supplementary Table 8 (g): Polyp Detection Rate (PDR) results stratified by center.

CONSORT-AI checklist of information to include when reporting a randomised trials of AI interventions

Section	Item	CONSORT 2010 Item[a]		CONSORT-AI Item	Addressed on Page No[b]
Title and Abstract					
**Title and Abstract**	1a	Identification as a randomised trial in the title	CONSORT-AI 1a,b Elaboration	(i) Indicate that the intervention involves artificial intelligence/machine learning in the title and/or abstract and specify the type of model.	1
	1b	Structured summary of trial design, methods, results, and conclusions (for specific guidance see CONSORT for abstracts)		(ii) State the intended use of the AI intervention within the trial in the title and/or abstract.	1 + 2
Introduction					
**Background and objectives**	2a	Scientific background and explanation of rationale	CONSORT-AI 2a (i) Extension	Explain the intended use of the AI intervention in the context of the clinical pathway, including its purpose and its intended users (e.g. healthcare professionals, patients, public).	4
	2b	Specific objectives or hypotheses			4
Methods					
**Trial design**	3a	Description of trial design (such as parallel, factorial) including allocation ratio			11
	3b	Important changes to methods after trial commencement (such as eligibility criteria), with reasons			11 - 13
**Participants**	4a	Eligibility criteria for participants	CONSORT-AI 4a (i) Elaboration	State the inclusion and exclusion criteria at the level of participants.	11 – 13
			CONSORT-AI 4a (ii) Extension	State the inclusion and exclusion criteria at the level of the input data.	11 – 13
	4b	Settings and locations where the data were collected	CONSORT-AI 4b Extension	Describe how the AI intervention was integrated into the trial setting, including any onsite or offsite requirements.	10 - 11
**Interventions**	5		CONSORT-AI 5 (i) Extension	State which version of the AI algorithm was used.	10

		The interventions for each group with sufficient details to allow replication, including how and when they were actually administered	CONSORT-AI 5 (ii) Extension	Describe how the input data were acquired and selected for the AI intervention.	10 - 11
			CONSORT-AI 5 (iii) Extension	Describe how poor quality or unavailable input data were assessed and handled.	10 - 11
			CONSORT-AI 5 (iv) Extension.	Specify whether there was human-AI interaction in the handling of the input data, and what level of expertise was required of users.	10 - 11
			CONSORT-AI 5 (v) Extension	Specify the output of the AI intervention	10 - 11
			CONSORT-AI 5 (vi) Extension	Explain how the AI intervention's outputs contributed to decision-making or other elements of clinical practice.	10 - 11
**Outcomes**	6a	Completely defined pre-specified primary and secondary outcome measures, including how and when they were assessed			12 - 13
	6b	Any changes to trial outcomes after the trial commenced, with reasons			N/A
**Sample size**	7a	How sample size was determined			12 - 13
	7b	When applicable, explanation of any interim analyses and stopping guidelines			N/A
Randomisation					
**Sequence generation**	8a	Method used to generate the random allocation sequence			11
	8b	Type of randomisation; details of any restriction (such as blocking and block size)			11
**Allocation concealment mechanism**	9	Mechanism used to implement the random allocation sequence (such as sequentially numbered containers), describing any steps taken to conceal the sequence until interventions were assigned			11
**Implementation**	10	Who generated the random allocation sequence, who enrolled participants, and who assigned participants to interventions			11

**Blinding**	11a	If done, who was blinded after assignment to interventions (for example, participants, care providers, those assessing outcomes) and how			11
	11b	If relevant, description of the similarity of interventions			NA
**Statistical methods**	12a	Statistical methods used to compare groups for primary and secondary outcomes			13
	12b	Methods for additional analyses, such as subgroup analyses and adjusted analyses			13
Results					
**Participant flow** (a diagram is strongly recommended)	13a	For each group, the numbers of participants who were randomly assigned, received intended treatment, and were analysed for the primary outcome			11
	13b	For each group, losses and exclusions after randomisation, together with reasons			4 + Figure 3
**Recruitment**	14a	Dates defining the periods of recruitment and follow-up			4
	14b	Why the trial ended or was stopped			N/A
**Baseline data**	15	A table showing baseline demographic and clinical characteristics for each group			Supplementary Table 3
**Numbers analysed**	16	For each group, number of participants (denominator) included in each analysis and whether the analysis was by original assigned groups			4
**Outcomes and estimation**	17a	For each primary and secondary outcome, results for each group, and the estimated effect size and its precision (such as 95% confidence interval)			4 – 5
	17b	For binary outcomes, presentation of both absolute and relative effect sizes is recommended			4 – 5
**Ancillary analyses**	18	Results of any other analyses performed, including subgroup analyses and adjusted analyses, distinguishing pre-specified from exploratory			4 – 5

**Harms**	19	All important harms or unintended effects in each group (for specific guidance see CONSORT for harms)	CONSORT-AI 19 Extension	Describe results of any analysis of performance errors and how errors were identified, where applicable. If no such analysis was planned or done, explain why not.	4 - 5
Discussion					
**Limitations**	20	Trial limitations, addressing sources of potential bias, imprecision, and, if relevant, multiplicity of analyses			7 - 9
**Generalisability**	21	Generalisability (external validity, applicability) of the trial findings			6 – 10
**Interpretation**	22	Interpretation consistent with results, balancing benefits and harms, and considering other relevant evidence			6 - 10
Other Information					
**Registration**	23	Registration number and name of trial registry			11
**Protocol**	24	Where the full trial protocol can be accessed, if available			N/A
**Funding**	25	Sources of funding and other support (such as supply of drugs), role of funders	CONSORT-AI 25 Extension.	State whether and how the AI intervention and/or its code can be accessed, including any restrictions to access or re-use.	14

[a] We strongly recommend reading this statement in conjunction with the CONSORT 2010 Explanation and Elaboration for important clarifications on all the items.

[b] Indicates page numbers to be completed by authors during protocol development
